# Three types of passivators on the stabilization of exogenous lead-contaminated soil with different particle sizes

**DOI:** 10.1038/s41598-021-01685-6

**Published:** 2021-11-19

**Authors:** Shuai Zhao, Xiongfei Cai, Ji Wang, Ding Li, Shijie Zhao, Xinjie Yu, Die Xu, Shuai Zhang

**Affiliations:** 1 College of Geography and Environmental Science, Guizhou Normal University, Guiyang, 550025 China; 2 The State Key Laboratory Incubation Base for Karst Mountain Ecology Environment of Guizhou Province, Guiyang, 550025 China

**Keywords:** Pollution remediation, Environmental impact, Environmental sciences, Environmental social sciences

## Abstract

Study on the form partitioning and content of heavy metals in soil particles with different sizes is crucial for preventing and controlling heavy metals pollution, but few studies regard soil contaminated by heavy metals as a homogeneous body. In this study (Fig. 1), goat manure, lime and phosphate were used to stabilize exogenous lead (Pb). These soil passivators’ differential effects on total Pb and Pb with different chemical forms in soil particles of different sizes as well as Pb immobilization in soil were investigated. By passivation experiment in laboratory for 45 days, the passivation effect of the single and combined application treatments on exogenous Pb and partitioning characteristics were analyzed and compared. The characterization method of fine sand microstructure and mineral composition analysis was used. The results showed that the single application of P5 and combined application of LP5 had optimum passivation efficiency. The content of DTPA-Pb was reduced with P5 by 65.27% and the percentage of available Pb decreased significantly in soil particles of the four sizes. The content of TCLP-Pb and available Pb (weak acid extraction and reducible Pb) significantly decreased by 71.60 and 25.12% respectively after the application of LP5 in the original soil. Furthermore, most of the total Pb was enriched in coarse sand and clay, while its content was lower in fine sand and silt. The combined application treatment of GL5 significantly increased the content of weak acid extractable and reducible Pb in fine sand, silty sand and clay. Through SEM and XRD analysis, it was found that the diffraction peak of P5 treatment groups might be related to the formation of insoluble Pb that contained compounds, which were mainly mineral components, including quartz, feldspar and mica, and LP showed a big potential in the study on passivation of heavy metal Pb-contaminated soil in the natural environment. In conclusion, further studies on the different dosage and metal-contamination levels as well as different combination forms of passivators should be considered under natural conditions, the selection of suitable passivators according to soil texture is of great significance for remediation of Pb-contaminated soil.

## Introduction

Soil is the material basis and an indispensable natural resource that human beings depend on for survival. With the rapid advancement of industrialization and urbanization, conflicts of soil pollution from heavy metal have become increasingly prominent. As a highly accumulated heavy metal element in soil, lead (Pb) comes from a wide range of sources and causes great ecological harm. Emissions of “three wastes”, unreasonable application of pesticide and fertilizers as well as mining and smelting activities all cause Pb pollution of soil, and the most serious pollution is caused in the mining and smelting process of lead–zinc ore^1^.

Partitioning of Pb in soil particles with different sizes is not uniform, and Pb is preferentially adsorbed at the surface of small soil particles^2^. Study has shown that the finerthe soil particles are, the stronger the enrichment ability of heavy metals will be^3^. At the same time, fine soil particles are more likely to migrate under the action of colloid co-migration, resulting in pollution of other environmental media^4,5^. In addition, soil physicochemical properties make the partitioning of chemical activities of Pb highly uneven, which leads to a great difference in the Pb absorption efficiency of organisms^6^. Nowadays, there are many remediation technologies for heavy metals contaminated soil. Conventional techniques for soil remediation include washing^7^, Phytoremediation^8^, immobilization^9^, and thermal treatment^10^. Application scopes and remediation effects of different methods arealso varied^11^. In situ chemical passivation can better meet the remediation requirements of heavy metals contaminated soil in terms of remediation time and economic costs, and the passivation effect and mechanism have been widely studied. Organic materials, lime and phosphate are cheap with wide sources and excellent passivation effects on heavy metals^12^. Organic materials lower the availability of heavy metals through adsorption, complexation/chelation, redox, etc., and they also indirectly reduce the harm of heavy metals by affecting the physicochemical properties of soil as well as the abundance and activities of soil microorganisms^13,14^. Lime reduces heavy metal availability by promoting concentrations of Pb^2+^ to form Pb(OH)_2_ and PbCO_3_ deposits through increasing soil pH^15^. Phosphate and Pb can generate phosphate precipitates and form very stable phosphor lead with halogen (Cl^−^, F^−^) in the soil. Passivation mechanism of lime and phosphate is relatively simple^16^. However, soil is not a homogeneous body, and the partitioning of different matter components in soil particles with different sizes is not uniform. Many specific reactions or phenomena only occur within a specific range of soil particle sizes^17^. Hence, the transformation and enrichment of heavy metals in soil with different particle sizes is very important for the remediation effect of passivators. Although there are many studies on chemical passivation remediation of Pb-contaminated soil, most of them regard soil as a homogeneous body. There are few studies on the migration and transformation of Pb in soil with different particle sizes after passivators are added into soil, and the internal microscopic mechanism of passivator remediation in Pb-contaminated soil is still not clear. The contents of three forms (DTPA-Pb, TCLP-Pb and the fractions of the sequential extraction) were used to reflect the stabilizing effect of different passivators on Pb-contaminated soil. The distribution of Pb in contaminated soil was explored by calculating the total Pb and forms of Pb in soil particles with different particle sizes, which has certain reference value for improving heavy metal contaminated soil.

**Figure 1:**
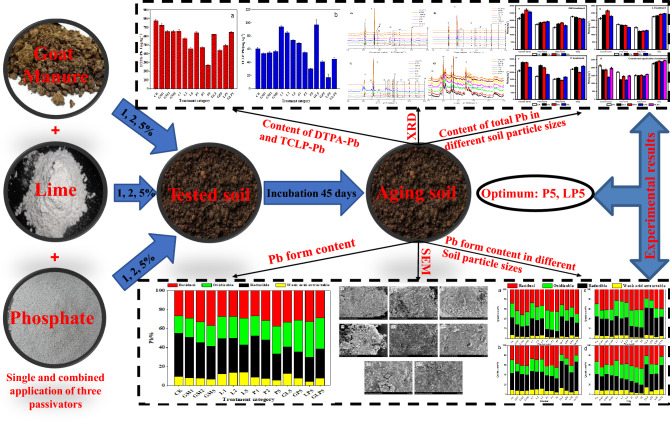
Overview of the main content of the study.

Therefore, the objectives of this study are: (1) to compare the effects of different passivators on the stabilization of exogenous-Pb contaminated soil; (2) to analyze the influence of different passivators on Pb enrichment and form partitioning in soil with different particle sizes; (3) to analyze and discuss micromorphology of different soil particles by the characterization method; (4) to assess the optimum type of potential soil passivators for Pb immobilization in exogenous-Pb contaminated soil (Fig. 1).

## Materials and methods

### Tested materials

The tested sample was taken from a vegetable field in Huaxi District, Guiyang City, Guizhou Province (106°39′48″E, 26°21′20″N), China, and the soil type was yellow soil. Topsoil (0–20-cm) was selected, and its physicochemical properties are shown as Table 1. The tested moist soil sample was air-dried at room temperature in the laboratory, and then it passed through a 2-mm mesh sieve. Pb(NO_3_)_2_ solution was used as the source of Pb contamination, and after adding it into the tested soil, the concentration of Pb^2+^ in the tested soil reached 2000 (mg kg^−1^). With the weighing method, ultrapure water was added to keep the moisture content of the soil sample at 60%. After 45 days’ incubation followed by air-drying, the soil was ground and then it passed through a 2-mm mesh sieve before use.

**Table 1:** Physicochemical properties of soil.

Soil parameter	Measured result
Soil pH	6.46
Field capacity/%	30.40
Cation exchange capacity (CEC) (cmol kg^−1^)	31.30
Soil organic matter (SOM) content (g kg^−1^)	36.89
Total nitrogen (g kg^−1^)	2.20
Total phosphorus (g kg^−1^)	0.58
Total kalium (g kg^−1^)	12.46
Rapidly available phosphorus (g kg^−1^)	41.32
Total Pb (g kg^−1^)	84.58

### Passivators

Goat manure (GM), Lime (Ca(OH)_2_) and Phosphate (Ca(H_2_PO_4_)·2H_2_O) were the three types of passivators used in the study. Goat manure (GM) was collected from a goat farm in Xiuwen, Guizhou Province, and its physicochemical properties are shown in Table 2. Ca(OH)_2_ (L) and Ca(H_2_PO_4_)·2H_2_O (P) were purchased from Upright and Zhiyuan Chemical Reagents Co., Ltd, China with guaranteed reagent.

**Table 2:** Physicochemical properties of the goat manure.

Property	Value
pH	7.27
Ash content	57.53%
Pb	25.77 mg/kg
Fe	1.46% (weight percentage)
C:N	12.29
H	4.05%
S	0.61%
O	30.43%
P	1.13 g/kg

### Experimental design

Passivation experiments were categorized as single and combined applications with a total of thirteen treatment groups. Each treatment group was repeated three times and was added as control check (CK) successively. Single applications: application rates of GM, L, and P were 1, 2, and 5% respectively. Combined applications: mixing GM, L, and P by a ratio of 1:1 with application quality equaling 5% of the soil quality (GL5, GP5, LP5, and GLP5) (Table 3). 300 g Pb-contaminated soil was put into the 500 mL experimental pot. Soil with passivators of the proportions mentioned above was mixed evenly. Soil moisture content was maintained at 60%, and then after 45 days’ incubation, the soil was dried, ground and reserved for use.

**Table 3:** Treatment category and application ratio.

Application type	Combination of passivator	Application rate/%		
Single applications	GM	1	2	5
	L	1	2	5
	P	1	2	5
Combined applications	GL	5		
	GP	5		
	LP	5		
	GLP	5		

Combined applications: mixing GM, L, and P by a ratio of 1:1.

### Classification of different soil particle sizes

**Figure 2:**
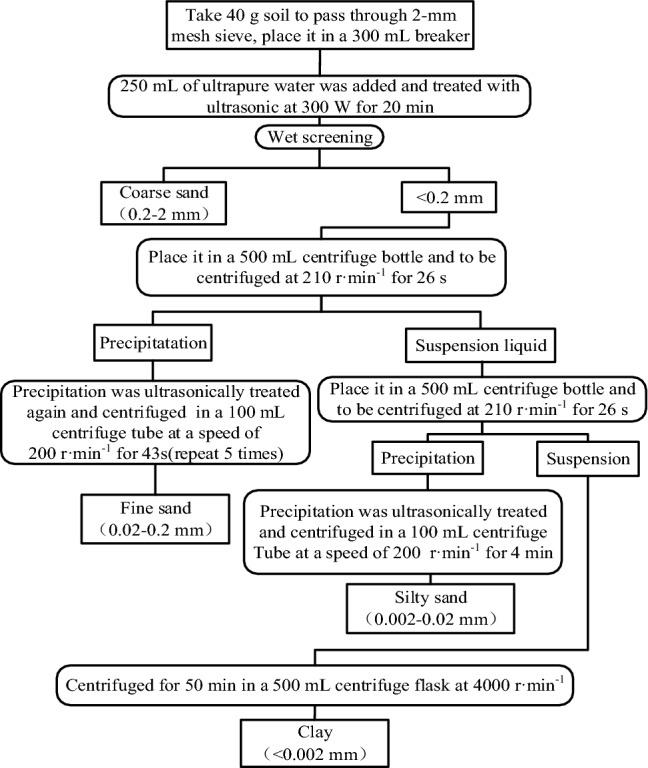
Separation process of soil with different particle sizes (Microsoft Office Visio).

The research method referred to^18^, separation process of soil with different particle sizes shown in Fig. 2. The samples passing through a 2 mm mesh sieve were firstly treated by ultrasound, and then coarse sand particles (0.2–2 mm) were separated by the wet sieve method. Finally, according to the Stokes formula, fine sand particles (0.02–0.2 mm), silt particles (0.002–0.02 mm) and clay particles (< 0.002 mm) were separated by the centrifugal method successively. In order to ensure the accurate separation of particle sizes, the size partitioning of soil particles was verified immediately after the separation by a laser particle size analyzer (Mastersizer2000, Malvern, UK). The soil with different particle sizes was dried at 50℃, and then it passed through a 0.149 mm mesh sieve before use.

The time required for centrifugation can be obtained by the Stokes formula:

1 $$ t = \frac{{63.0 \times 10^{8} n\log (R/S)}}{{N^{2} D^{2} \Delta s}} $$

where *n* is the viscosity of soil suspension at experimental temperature and *R* (cm) is the distance between the precipitation and the centrifugal axis. *S* (cm) is the distance between the suspension surface and the centrifugal axis during centrifugation, *N* (r min^−1^) is the centrifuge speed, *D* (μm) is the diameter of soil particles, and △*s* is the gravity acceleration difference between the suspended soil particles and the surrounding liquid. Since the part < 2-μm was not classified in this study, it was set as 1.653.

### Analytical methods

Soil pH, Organic matter, cation exchange capacity, total nitrogen, total phosphorus and rapidly available P reference^19^. pH of the soil samples was determined in a water-soil suspension (2.5:1) using a glass electrode pH meter (Model PHS-3C^+^, Shanghai INESA Co. Ltd.), and SOM content was determined with the K_2_Cr_2_O_7_ volumetric method. CEC was determined by cobalt hexamine trichloride extraction-spectrophotometry. Total N and P as well as rapidly available P were measured by an automatic discontinuous chemical analyzer (CleverChem200+, Germany DeChem-Tech Gmbh Co., Ltd). Total Pb and K content was measured by HCl, HNO_3_, HF, HClO_4_ digestion. The available Pb (DTPA-Pb) was analyzed by the DTPA (diethylenetriamine pentaacetic acid) extraction method (Chinese GB/T 23739-2009). The TCLP-Pb was analyzed by CH_3_COOH^20^. Chemical forms of Pb in soil were measured by the improved continuous extraction method of BCR^21^. The content of Pb and K was analyzed with an atomic absorption spectrometer (GGX-800, Beijing Haiguang Instrument Co., Ltd).

### Statistical analysis

Statistical analysis of raw data in the study was collated and calculated by Microsoft Excel 2019. Experimental data presented herein was the mean of the three samples. Standard deviation of the mean was indicated by the error bars. The Pearson correlation matrix analysis was treated by SPSS 22.0 software. The experimental images were plotted by Origin 2019 and Microsoft PowerPoint.

## Results and discussion

### Effect of soil passivators on Pb form

**Figure 3:**
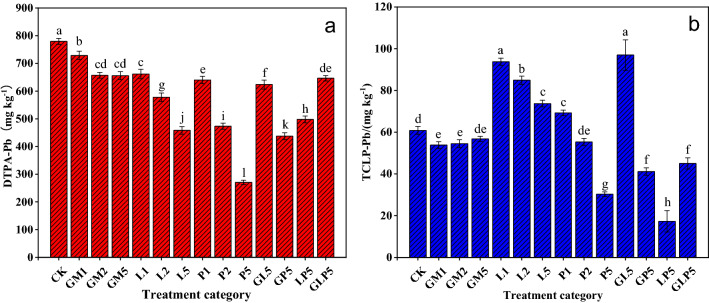
Content of DTPA-Pb and TCLP-Pb under different passivators (Origin 2019b). (CK: control check, GM: goat manure, L: Ca(OH)_2_, P: Ca(H_2_PO_4_)·2H_2_O, GL: goat manure + Ca(OH)_2_, GP: goat manure + Ca(H_2_PO_4_)·2H_2_O, LP: Ca(OH)_2_ + Ca(H_2_PO_4_)·2H_2_O, GLP: goat manure + Ca(OH)_2_ + Ca(H_2_PO_4_)·2H_2_O; 1, 2, 5 represents the proportion of passivators).

For farmland soil, the uptake of heavy metals by crops is directly related to the quality of agricultural products, the choice of evaluation methods are more inclined to evaluate plant can give sex, DTPA usually can extract heavy metal water soluble form, exchangeable form, the sum of the organic combination form, also includes some oxides and secondary clay minerals content of heavy metal, because they most closely, and plant growth Plant availability that best represents heavy metals^22^. DTPA-Pb is considered one of the bioavailable or labile pools which have highly potential ecotoxicity on the environment^23^. It has been revealed that Pb in plants was significantly related with DTPA-extractable fractions in soils^24^. The passivation effect was enhanced as the dosage of passivators in single application treatment groups increased. The DTPA-Pb concentration significantly decreased by 123.86 (mg kg^−1^), 321.41 (mg kg^−1^) and 508.93 (mg kg^−1^) respectively after the application of GM5, L5 and P5 for 45 days (Fig. 3a). Among all the treatment groups, P5 had a better passivation effect (decreased by 65.27%), while GM1 had the weakest effect (decreased by 6.61%) on DTPA-Pb content. By comparing and analyzing the differences in stability of Pb(OH)_2_ and Pb_3_(PO_4_)_2_, it was found that the passivation effect of phosphate was better than that of alkaline materials^25^. In some cases, it’s been reported that L has a better passivation effect on heavy metals than organic materials do on the acidic soil, which is similar to this study^26^. However, this is closely related to the physical and chemical properties of soil and the application rate. Toxicity Characteristic Leaching Procedure (TCLP) was created after the U.S. EPA Method 1311 (USEPA 1992) with minor modifications^27^. It is one of the most commonly used ecological risk assessment methods to detect the dissolution and migration of heavy metals in solid media or waste. As it is shown in Fig. 3b, the TCLP-Pb content was significantly different in the treatment groups. The application of GM could reduce the TCLP-Pb content in the tested soil, and the decrease was inversely proportional to its dosage. But, the application of L significantly increased the TCLP-Pb content, and the TCLP-Pb content decreased with the increase of L dosage. Among all the treatment groups, LP5 was the most effective in all the treatment groups, and TCLP-Pb content significantly decreased by 71%. GL5 had the least effect, increasing TCLP-Pb content by 59%. On the whole, P5, GP5, and LP5 treatment groups had the best effect on DTPA-Pb and TCLP-Pb. However, application of L significantly had increased the TCLP-Pb content in this study. Lower levels of lime treatment (1%, 2% and 5%) increased the contents of TCLP-Pb and leachability of Pb in soil due to the pH reduction. The lime induced the formation of the C-S-H and ettringite. Reduction of the TCLP-Pb might result from complexation of Pb on the surface of the formed calcite. High pH would enhance adsorption of Pb on the calcite surface. Immobilization may also be associated with the formation of calcium silicon hydrates and calcium aluminum hydrates and ettringite with the addition of lime by sorption, phase mixing or substitution^28^. In general, the effectiveness of the quicklime treatments is closely related to the physical and chemical properties of soil. One-way ANOVA was used to analyze the significant differences in DTPA-Pb content under different passivator treatments. In the single application group, which highlights differences in DTPA-Pb extraction ability according to their dosages, this phenomenon is most obvious in phosphate treatment. The DTPA-Pb content in P5 treatment was significantly lower than that in other treatment groups, which is similar to TCLP-Pb content under different passivator treatments. However, its worth noting that the passivation effect of TCLP-Pb in the combined application group is the most significant, which is different from the extraction morphology of DTPA-Pb.

### Effect of soil passivators on Pb form partitioning

**Figure 4:**
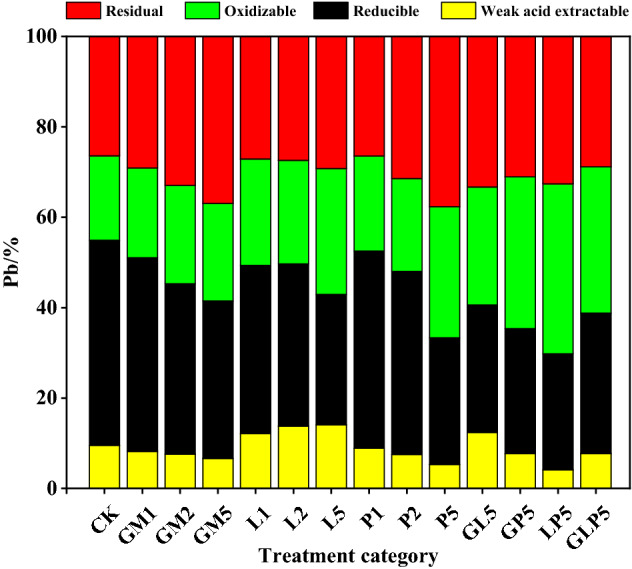
Effects of different passivators treatment on the form of Pb (Origin 2019b). (CK: control check, GM: goat manure, L: Ca(OH)_2_, P: Ca(H_2_PO_4_)·2H_2_O, GL: goat manure + Ca(OH)_2_, GP: goat manure + Ca(H_2_PO_4_)·2H_2_O, LP: Ca(OH)_2_ + Ca(H_2_PO_4_)·2H_2_O, GLP: goat manure + Ca(OH)_2_ + Ca(H_2_PO_4_)·2H_2_O; 1, 2, 5 represents the proportion of passivators).

The sequential extraction of heavy metals in soils was carried out following the modified European Community Bureau of reference sequential extraction procedure^29^. Chemical forms of heavy metals in soil cause different degrees of threat to the ecological environment. Passivators can reduce the harm of heavy metals to organisms and ecological environment by changing the occurrence form of heavy metals. Therefore, form classification scheme can be used to evaluate the passivation effect of Pb-contaminated soil. The content percentage of Pb chemical forms can directly reflect the influence of passivators on the partitioning of Pb forms in Pb-contaminated soil (Fig. 4). In the original soil, Pb was mainly present in weak acid extractable, reducible, and oxidizable forms, and the sum of the three forms was about 73.56%. Research results showed that both LP5 and P5 treatment groups significantly reduced the percentage of weak acid extractable and reducible Pb in Pb-contaminated soil, which were 21.54% and 25.12% lower than that of the CK treatment groups (without soil passivators). The GM and L treatment groups reduced the content of available Pb, and the decrease was positively correlated with the applied dosage of passivators. Application of L in the tested soil increased the soil pH, contributing to increased content of weak acid extractable Pb, because the concentration of Pb(OH)_2_ and Pb_2_CO_3_ increased by L. All treatment groups could reduce the content of reducible Pb in the tested soil and the effect of single treatments in a descending order was brought by GM2, L5 and P5. Among the treatment groups, oxidizable Pb with GM, L, and P decreased by 1.17–5.44%, 4.22–14.19%, and 2.35–17.89% respectively. Both the GM and P treatment groups increased residual Pb content, and P5 had the optimum passivation effect, Pb immobilization may be attributed to the P-induced conversion of Pb from soluble cerussite to insoluble Pb phosphate minerals^30,31^. while the L treatment groups had little effect on the residual Pb content. GL5 increased the content of weak acid extractable Pb by 10.45%, the results showed that the combination of sheep manure and lime had poor passivation effect, which increased the content of active Pb in tested soil. But GP5, LP5 and GLP5 decreased the content of weak acid extractable Pb in the passivator treatment groups, it shows that phosphate play an obvious role in combined application treatment. DTPA extractant is made up of (Diethylenetriamine pentaacetic acid (DTPA), Triethanolamine (TEA) and CaCl_2_·2H_2_O) was used to extract the Pb in soil, there was a high correlation between their content and the absorption of Pb by crops. Acetic acid was used as the extractant of TCLP-Pb, the TCLP-Pb test is designed to determine the mobility of Pb-contaminants in soil^32^. Acetic acid, hydroamine hydrochloride, hydrogen peroxide and ammonium acetate were used as extractants for sequential extraction of Pb, the Pb can be divided into four different extraction forms by availability classification, and their bioavailability decreases with the decrease of activity. The three extraction methods have great differences, mainly because of the different kinds of extractants used, and different experimental conditions have obvious differences on the extracted Pb content.

### Total Pb content in soil particles with different sizes

**Figure 5:**
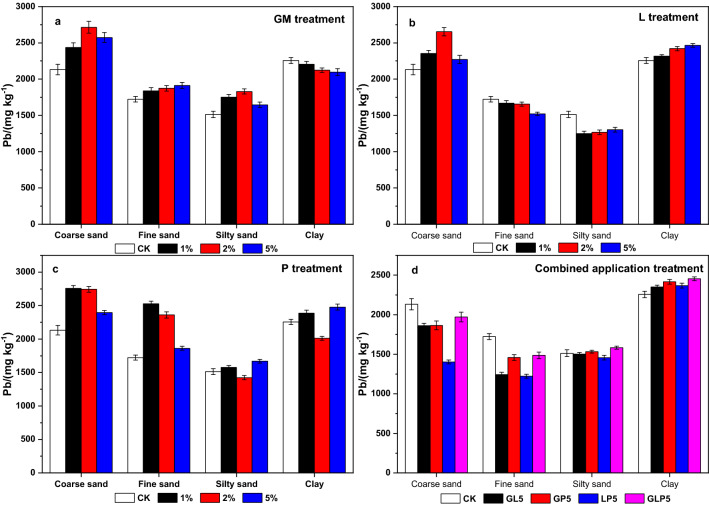
Content of total Pb in soil of different particle sizes under passivators (Origin 2019b).

The content partitioning of total Pb in particle sizes under different kinds of passivators and applying doses are shown in the Fig. 5. Total Pb content was a dual peak distribution in soil of different particle sizes. Specifically, the total Pb content in coarse sand and clay was higher, while in fine sand and silty sand, it was lower. The cause of Pb enrichment in coarse sand is complicated. Some studies believe that the SOM and other substances are enriched on the surface of coarse sand particles after agglomeration by the compound action of Pb^33^, while believe that coarse sand contains coarse minerals or heavy minerals with strong retention ability of heavy metals^34^. The specific surface area of clay particles is larger, the content of clay minerals and Fe–Mn/Fe–Al oxides is higher, and the adsorption capacity of total Pb is greater. Total Pb content in coarse sand, fine sand and clay soil in the GM treatment groups increased by different degrees, and the effect by GM2 was the most significant. Total Pb content in coarse sand and clay increased by 583 (mg kg^−1^) and 317 (mg kg^−1^) respectively, which might be caused by the higher content of Pb in the GM. GM5 could increase the content of total Pb in fine sand, and through passivation by GM, clay in the Pb level dropped, probably because after applying GM, the SOM content was promoted. However, different particle sizes of SOM in the activity had different effects with sand, silt and clay in a descending order^35^. In general, higher content of SOM could improve adsorption capacity of pollutants, negative charge on the surface of soil particles improved with the increase of L dosage, and the cementing materials of CaCO_3_ and Ca(OH)_2_ were formed. The large specific surface area of clay particles brought a high quantity of negative charge and strong adsorption capacity for Pb^2+^. The cementing materials in coarse sand particles made exchanges and adsorbed Pb^2+^ to increase Pb retention. Compared with CK, enrichment degree of Pb increased in coarse and fine sand after P treatments, but the content of Pb in silty sand and clay particles was not significantly affected. Combined application treatments of GL5, GP5, LP5 and GLP5 reduced the content of Pb in coarse and fine sand, but it had little effect on the Pb content in the silt and increased the total Pb content in the clay particles which was up to 110.93 (mg kg^−1^), which might be caused by the interactions of different passivators during immobilization.

### Content of different forms of Pb in soil with different particle sizes

**Figure 6:**
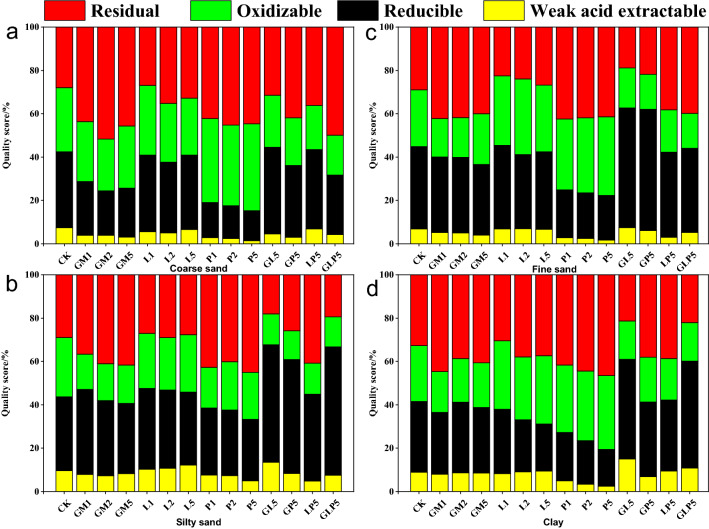
Effect of passivators on fractions of Pb in soil with different particle sizes (Origin 2019b). (CK: control check, GM: goat manure, L: Ca(OH)_2_, P: Ca(H_2_PO_4_)·2H_2_O, GL: goat manure + Ca(OH)_2_, GP: goat manure + Ca(H_2_PO_4_)·2H_2_O, LP: Ca(OH)_2_ + Ca(H_2_PO_4_)·2H_2_O, GLP: goat manure + Ca(OH)_2_ + Ca(H_2_PO_4_)·2H_2_O; 1, 2, 5 represents the proportion of passivators).

Redistribution of Pb in soil with different particle sizes was affected by passivators, which might also change the form partitioning of Pb in each particle size, thus affecting the availability of Pb in soil. As it can be seen from Fig. 6, there was no significant difference in the form partitioning of Pb in all soil particle sizes under the CK treatment, indicating that there was no significant difference in the partitioning trend of exogenous Pb in all soil particle sizes after it entered the soil^36^. It is worth mentioning that P5 treatment with other particle sizes had a better stabilization effect on exogenous Pb contaminated soil, in which the percentage of weak acid extractable and reducible Pb with high bioavailability was significantly reduced. The reason might be that the smooth coating formed by soluble phosphate and other mineral crystals made Pb^2+^ trapped on the surface of soil particles^37^. In general, in the treatment groups of combined application, the percentage of oxidizable and residual Pb decreased significantly, while the percentage of weak acid extractable and reducible Pb with high availability increased, and the passivation effect was not strong.

### Properties of the soil particles

#### Morphology

**Figure 7:**
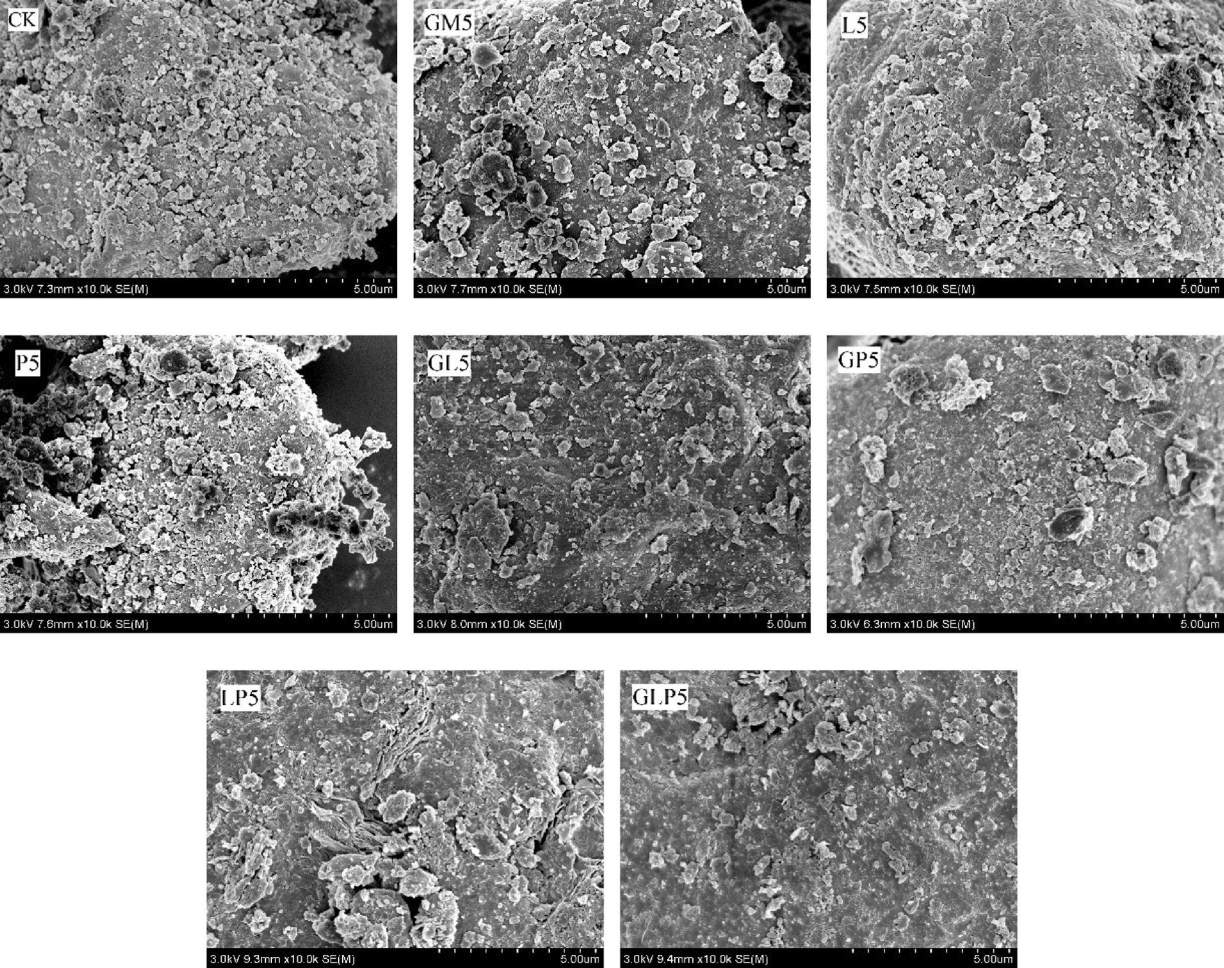
SEM images of fine sand soil particles before and after passivator treatments. (CK: control check, GM: goat manure, L: Ca(OH)_2_, P: Ca(H_2_PO_4_)·2H_2_O, GL: goat manure + Ca(OH)_2_, GP: goat manure + Ca(H_2_PO_4_)·2H_2_O, LP: Ca(OH)_2_ + Ca(H_2_PO_4_)·2H_2_O, GLP: goat manure + Ca(OH)_2_ + Ca(H_2_PO_4_)·2H_2_O; 1, 2, 5 represents the proportion of passivators).

The morphology and microstructure of the soil samples were observed by SEM (SU8020, Hitachi, Japan). SEM was used to scan and photograph the surface of fine sand both before and after the passivation treatment, and the magnification was 5000 times. As it can be seen Fig. 7, after the passivation treatment, the micromorphology of soil particles changed as follows: GM increased the amount of large particle matters over 10 mm on the surface of fine sand grains, and the surface smoothness increased after the L treatment, but the small particles decreased obviously on the surface. The P treatment resulted in protruding structures on the surface of soil particles. In the combined application treatment of GP5, the surface of soil particles was smooth and fine particles were greatly reduced, but the large particles over 10-mm increased significantly. After the application of LP5, the surface of soil particles wrinkled deeply, furrows and protrusions appeared, and large particles over 15 mm came up on the surface. The fine particles of the surface decreased and the surface structure was relatively flat, but the surface sagged after the application of GLP5.

#### XRD

**Figure 8:**
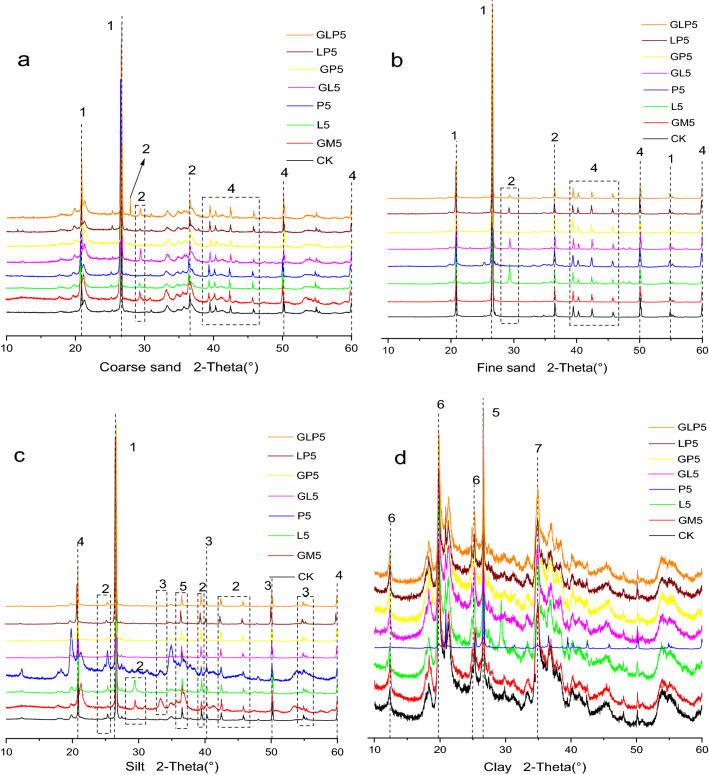
XRD patterns of coarse sand, fine sand, silt, and clay soil particles after passivator treatments. (1: quart, 2: feldspar, 3: mica, 4: zeolite, 5: illite, 6: chlorite, 7: calcite) (Origin 2019b). (CK: control check, GM: goat manure, L: Ca(OH)_2_, P: Ca(H_2_PO_4_)·2H_2_O, GL: goat manure + Ca(OH)_2_, GP: goat manure + Ca(H_2_PO_4_)·2H_2_O, LP: Ca(OH)_2_ + Ca(H_2_PO_4_)·2H_2_O, GLP: goat manure + Ca(OH)_2_ + Ca(H_2_PO_4_)·2H_2_O; 1, 2, 5 represents the proportion of passivators).

The crystalline structures of the soil samples were characterized by an X-ray diffractometer (Brook D8 Advance, Brook GmbH, Germany). Minerals such as quart, feldspar, mica, zeolite, illite, chlorite and calcite were mainly found in the simulated Pb-contaminated soil particles (Fig. 8), the content of quartz in coarse sand, fine sand and silt was high, accompanied by low content of feldspar, mica, zeolite and illite. Main components of clay particles were illite and chlorite^38,39^. With the decrease of soil particle sizes, clay minerals gradually increased. The characteristic peak of feldspar in coarse sand showed differences in several passivator treatment groups (around 30°). The peak appeared at d = 0.319 nm (2θ = 27.89°) in the GLP5 treatment, and the reason might be that Pb-Ca mixed phosphate and Pb_3_(PO_4_)_2_^31^. Another diffraction peak appeared at d = 0.324 nm (2θ = 27.43°) in several treatments, which might be caused by the presence of PbSO_3_ in coarse sand particles. All the passivation treatment groups containing L in fine sand showed an obvious diffraction peak, indicating that the application of L could induce the formation of PbSi_2_O_7_ in fine sand. In addition, Pb_3_O_2_Cl_2_ diffraction peaks appeared at d = 0.209 nm (2θ = 43.15°) and d = 0.187 nm (2θ = 48.49°) under L5 and GL5 treatments, and PbF_2_ appeared at d = 0.191 nm (2θ = 47.15°). The extra peak of L5 treatment at d = 0.303 nm (2θ = 29.40°) also appeared in the silty sand and clay, indicating that L could also induce the formation of Pb_3_Si_2_O_7_. The characteristic peak of feldspar appeared in GM, L, GL5 and GLP5, because under the application, GM and L contained calcium minerals. The reason that P treatment group had no characteristic peaks might be that some L and GM contained calcium components and had chemical reactions. The diffraction peak of P treatment group was different from that of other treatment groups, the burr peak increased, and the sharp peak was also different from that of other treatment groups. Sand soil was usually composed of large soil particles with high quartz content and primary minerals such as feldspar, mica, and zeolite, which broke into fine particles under weathering. Coarse sand, fine sand and silt contained more crystalline minerals, and XRD pattern was more similar. The peak followed rules. As the soil particle size decreased, fewer crystal minerals were broken. On XRD amorphous feature maps, there would be more burr peaks, and clay X-ray diffraction intensity was obviously weaker than that of fine sand and silt components, demonstrating that the dispersion of clay components was higher with less crystal shape.

## Conclusions

The P5 treatment could reduce the content of DTPA-Pb by 65.27%, while LP5 treatment could reduce the content of TCLP-Pb and available Pb by 71.60 and 25.12% respectively in Pb-contaminated soil. The two passivation treatment groups showed outstanding performance in this research. The total Pb was mainly enriched in coarse sand and clay, and its content in fine sand and silty sand was low. P5 could significantly reduce the content percentage of available Pb (weak acid extractable and reducible Pb) in different particle sizes, while the combined treatment groups could increase the content of weak acid extractable and reducible Pb, which might be caused by the interactions between passivators that increased the availability of Pb. Through SEM and XRD analysis, it was found that diffraction peaks of P5 treatment groups might be related to the formation of insoluble Pb-containing compounds, and the main mineral components included quartz, feldspar and mica. Future research should focus on the dosage of passivator, heavy metals pollution level and the suitable combination of passivators should be considered under natural conditions.

## Data Availability

The datasets used and/or analyzed during the current study are available from the corresponding author on reasonable request.
